# Depletion of Kinesin 5B Affects Lysosomal Distribution and Stability and Induces Peri-Nuclear Accumulation of Autophagosomes in Cancer Cells

**DOI:** 10.1371/journal.pone.0004424

**Published:** 2009-02-10

**Authors:** Carla M. P. Cardoso, Line Groth-Pedersen, Maria Høyer-Hansen, Thomas Kirkegaard, Elizabeth Corcelle, Jens S. Andersen, Marja Jäättelä, Jesper Nylandsted

**Affiliations:** 1 Center for Neuroscience and Cell Biology, Faculty of Medicine, University of Coimbra, Coimbra, Portugal; 2 Apoptosis Department and Centre for Genotoxic Stress Research, Institute of Cancer Biology, Danish Cancer Society, Copenhagen, Denmark; 3 Centre for Experimental Bioinformatics (CEBI), University of Southern Denmark, Odense, Denmark; UT MD Anderson Cancer Center, United States of America

## Abstract

**Background:**

Enhanced lysosomal trafficking is associated with metastatic cancer. In an attempt to discover cancer relevant lysosomal motor proteins, we compared the lysosomal proteomes from parental MCF-7 breast cancer cells with those from highly invasive MCF-7 cells that express an active form of the ErbB2 (ΔN-ErbB2).

**Methodology/Principal Findings:**

Mass spectrometry analysis identified kinesin heavy chain protein KIF5B as the only microtubule motor associated with the lysosomes in MCF-7 cells, and ectopic ΔN-ErbB2 enhanced its lysosomal association. KIF5B associated with lysosomes also in HeLa cervix carcinoma cells as analyzed by subcellular fractionation. The depletion of KIF5B triggered peripheral aggregations of lysosomes followed by lysosomal destabilization, and cell death in HeLa cells. Lysosomal exocytosis in response to plasma membrane damage as well as fluid phase endocytosis functioned, however, normally in these cells. Both HeLa and MCF-7 cells appeared to express similar levels of the KIF5B isoform but the death phenotype was weaker in KIF5B-depleted MCF-7 cells. Surprisingly, KIF5B depletion inhibited the rapamycin-induced accumulation of autophagosomes in MCF-7 cells. In KIF5B-depleted cells the autophagosomes formed and accumulated in the close proximity to the Golgi apparatus, whereas in the control cells they appeared uniformly distributed in the cytoplasm.

**Conclusions/Significance:**

Our data identify KIF5B as a cancer relevant lysosomal motor protein with additional functions in autophagosome formation.

## Introduction

Lysosomes are membrane-bound dynamic organelles that represent the final destination for endocytic, secretory and autophagic pathways [1]. The physiological importance of lysosomes is highlighted by a number of diseases resulting from defects in the lysosomal biogenesis and function [2]. On the contrary, the enhanced synthesis, trafficking and extracellular release of lysosomal proteases (cathepsins), are important hallmarks of malignancy and associate with the invasive and metastatic capacity of cancer cells [3], [4]. Interestingly, the lysosomal changes associated with immortalization and transformation of cancer cells also sensitize cancer cells to programmed cell death pathways involving lysosomal membrane permeabilization [5], [6]. Once triggered, lysosomal membrane permeabilization results in the release of cathepsins and other lysosomal hydrolases to the cytosol, where they can trigger the mitochondrial outer membrane permeabilization followed by caspase-mediated apoptosis [7], [8] or mediate caspase-independent programmed cell death [9]. Thus, the inhibition of lysosomal trafficking/exocytosis appears as a promising target for cancer therapy. It would not only inhibit the cathepsin-mediated invasion but also obstruct the general trafficking and possibly result in the accumulation of lysosomes destined for secretion and therefore further sensitize cancer cells to lysosomal cell death pathways. This hypothesis is supported by data showing that vincristine, a microtubule-destabilizing anti-cancer drug, not only inhibits lysosome trafficking but also induces a rapid increase in the volume of the lysosomal compartment followed by lysosomal leakage and cathepsin-dependent cell death [10].

Because drugs that disturb the microtubule network show high general toxicity, we speculated that a more specific interference with lysosome trafficking could result in anti-cancer strategies with fewer side effects. Accordingly, we wanted to identify and characterize motor proteins important for lysosome transport in cancer cells. Motor proteins utilizing the cytoskeleton as substrate for movement are divided into myosin motors that move along actin microfilaments and kinesin/dynein motors that use microtubules through the interaction with tubulin for their movement [11]. Motor proteins are powered by the hydrolysis of ATP and convert chemical energy into mechanical work enabling them to move cargo (vesicles, proteins and lipids) over long distances. Microtubule specific motors consist of two basic types of microtubule motors: plus-end motors and minus-end motors, depending on the direction in which they move along the filaments within the cell [12].

The truncated form of the ErbB2 receptor is frequently found over-expressed in breast cancer and its expression and activity correlates with increased invasiveness, motility and poor prognosis [13]. Accordingly, the ectopic expression of ΔN-ErbB2 in MCF-7 breast cancer cells renders them highly mobile and invasive [14] (Our unpublished observation). Prompted by the finding that the ΔN-ErbB2-induced invasive phenotype was associated with altered lysosomal trafficking and a several fold increase in the expression and activity of lysosomal proteases, we chose this model system to search for cancer relevant lysosomal motor proteins. We applied a quantitative proteomic analysis on purified lysosomes from ΔN-ErbB2 MCF-7 and control cells showing that some motor protein levels were significantly up-regulated following ΔN-ErbB2 induction. Interestingly, we found that ΔN-ErbB2 increased the expression of kinesin 5B (KIF5B), a motor protein implicated in lysosomal and mitochondrial transport [15], [16]. In line with this, KIF5B mRNA has been reported to be up-regulated in several types of cancer tissues including bladder cancer (GDS1479 record), advanced gastric cancer (GDS1210 record), squamous cell carcinoma (GDS2200 record), sporadic basal-like breast cancer and BRCA1-associated breast cancer (GDS2250 record) (Data obtained from NCBI: http://www.ncbi.nlm.nih.gov/sites/entrez; Gene Expression Omnibus). KIF5B is a N-kinesin (Plus-end motor) belonging to the super family of kinesin-1 molecular motor proteins that together with cytoplasmic dynein is responsible for microtubule-dependent transport of cargo in eukaryotic cells [17]. To elucidate the role of KIF5B in cancer cells we examined its function in various lysosomal pathways including the lysosomal cell death pathway, the resealing response after plasma membrane damage (exocytosis) and macroautophagy.

## Materials and Methods

### Cell culture and treatments

MCF-7, HeLa and U2OS cells originate from human breast carcinoma, cervix carcinoma and osteosarcoma, respectivly. MCF-7-eGFP-LC3 cell line is a single cell clone of MCF-7 cells expressing a fusion protein consisting of enhanced green fluorescent protein (eGFP) and rat LC3 [18]. MCF-7-ΔNErbB2 and MCF-7-pTRE cell lines are single cell clones of MCF-7 expressing the tetracycline transactivator transfected with pTRE-ΔNErbB2 and pTRE, respectively [14]. HeLa-LIMP1-eGFP cells are HeLa cells expressing eGFP-tagged lysosome integral membrane protein-1 (LIMP-1) [19] (kindly provided by Dr. J.P. Luzio, University of Cambridge). The cancer cells and their transfected variants were propagated in RPMI 1640 (Invitrogen) supplemented with 6% heat-inactivated fetal calf serum (FCS; Biological Industries) and penicillin-streptomycin. The medium of MCF-7-ΔNErbB2 and MCF-7-pTRE was further supplemented with 5 μg/ml tetracycline. To induce the ΔN-ErbB2 expression, tetracycline (5 μg/ml) was removed and the cells were washed 5 times in PBS before plating. All cells were kept at 37°C in a humidified air atmosphere at 5% CO_2_.

### Analysis of lysosome-associated proteins by mass spectrometry using stable isotope labeling with amino acids in cell culture (SILAC)

MCF-7-ΔNErbB2 and MCF-7-pTRE were grown in custom-synthesized RPMI 1640 medium with either normal lysine 12C614N2 (Lys0) or isotope labeled L-lysine 13C615N2 (Lys8) (Sigma-Isotec, St. Louis, MO) supplemented with 10% dialyzed foetal calf serum (Invitrogen) for at least 5 cell divisions to fully incorporate the labeled amino acids. Lysosomes were purified by Iron-Dextran (FeDex) fractionation according to a protocol published previously [20]. Briefly, cells (80–90×10^6^ in total) preincubated with FeDex (8 h) were lysed mechanically in a dounce homogenizer and the light membrane fraction was loaded on a MiniMachs column attached to a magnet (MACS Separator system, Miltenyi Biotec). Lysosomes trapped on the column were eluted in sucrose extraction buffer (250 mM sucrose, 20 mM Hepes, 10 mM KCl, 1.5 mM MgCl_2_, 1 mM EDTA, 1 mM EGTA, and 1 mM pefabloc, pH 7.5) by removing the column from the magnet and flush out lysosomes by a plunger. Lysosomes were dissolved and proteins separated by electrophoresis on NuPAGE Bis-Tris 4–12% gradient gels (Invitrogen) and stained with Comassie Blue. Gel slices were cut into small pieces and incubated with 12.5 ng/μl trypsin at 37°C overnight. The resulting peptides were analyzed by liquid chromatography (Agilent HP1100) combined with tandem mass spectrometry (LC MS/MS) using a linear ion-trap Fourier-transform ion-cyclotron resonance mass spectrometer (LTQ-FT-ICR, Thermo-Finnigan). Peak list were extracted using an in-house developed scripts (DTA-supercharge), combined for each gel slides, and used for protein database searches. Stringent criteria were required for protein identification in the International Protein Index database using the Mascot program (Matrix Science): at least two matching peptides per protein, a mass accuracy within 3 p.p.m., a Mascot score for individual peptides of better than 20, and a delta score of better than 5. MS-Quant (http://msquant.sourceforge.net/), an in-house developed software program was used to calculate peptide abundance ratio and to evaluate the certainty in peptide identification.

### siRNAs and transfections

Three siRNAs were designed to target KIF5B mRNA: 5′-CCAUCAUCAUACAAUGAGUCUGAAA-3′ (KIF5B-1), 5′-CGGCGACAAGUACAUCGCCAAGUUU-3′ (KIF5B-2), and 5′-CAUCUACCAGAAGGGAUCAAGACAA-3′ (KIF5B-3). All siRNAs were purchased from Invitrogen. In every siRNA experiment a control sample treated with the transfection agent alone and/or a KIF5B mismatch oligo, 5′-CGGAACACAUGGCUAAACCGGCUUU-3′ (MM), were included. MCF-7 and HeLa cells were transfected with 25 nM of siRNA applying oligofectamine (Invitrogen) as the transfection agent.

### Measurement of cell viability and microscopic analysis

Viable cells were measured by their ability to reduce the tetrazolium salt 3-(4,5-dimethylthiazole-2-y)-2,5-diphenyltetrasodiumbromide (MTT; Sigma) to a formazan dye detectable by spectrophotometric analysis in a VersaMax microplate reader (Molecular Devices Ltd., Wokingham, United Kingdom) as described previously [9]. Phase contrast pictures of cell lines were taken with an inverted Olympus IX-70 microscope connected to an Olympus DP70 digital camera. Time lapse microscopy was performed with a Carl Zeiss Axiovert 200M fluorescence microscope using MetaMorph software.

### Analysis of GFP-LC3 translocation

Autophagy was induced by incubating MCF-7-LC3-eGFP cells with 2.5 μM rapamycin (Sigma-Aldrich, St. Louis, MO, USA) for 24 h. The percentage of cells with eGFP-LC3 translocation into dots (a minimum of 100 cells/sample) was counted in eGFP-LC3 expressing cells fixed in 3.7% formaldehyde and 0.19% picric acid (vol/vol) applying Zeiss Axiovert 100 M Confocal Laser Scanning Microscope. Cells with ≥5 green cytosolic vesicles were considered positive.

### Measurement of enzyme activities

Caspase-3-like (DEVD-AFC, Enxzyme System Products), cysteine cathepsin (zFR-AFC, Enzyme System Products), acid phosphatase and ß-*N*-acetyl-glucosaminidase (NAG) activities were determined essentially as previously described [6], [9]. Briefly, the cytoplasmic fraction was extracted with 20–35 μg/ml digitonin and the total cellular fraction with 200 μg/ml digitonin and the rate of the appropriate substrate hydrolysis V_max_ was measured over 20 min at 30°C on a Spectramax Gemini fluorometer (Molecular Devices, Sunnyvale, CA, USA). Lactate dehydrogenase (LDH) activity of the cytosol determined by a cytotoxicity detection kit (Roche) was used as an internal standard.

### Immunoblot analysis and immunocytochemistry

Proteins were separated by SDS-PAGE and transferred to nitrocellulose membranes. Primary antibodies raised against KIF5B (SUK4 from Developmental Studies Hybridoma Bank (DSHB), University of Iowa) and ab5629 (from Abcam), lysosome-associated membrane protein-2 (LAMP-2; clone H4B4 from DSHB), GRP75 (SPA825 from Stressgene,), cathepsin B (Ab1 from Oncogene), p70 S6 kinase 1 (p70^S6K1^; #9202) and phospo-p70^S6K1^(#9206) (from Cell Signalling Technology), and glyceraldehyde-3-phosphate (GAPDH, Biogenesis, Poole, UK) followed by appropriate peroxidase-conjugated secondary antibodies from DAKO A/S (Glostrup, Denmark).

For immunocytochemistry, cells on coverslips were fixed using ice-cold methanol for 10 min or 3,7% formaldehyde for 30 min at 25°C. Cells were stained with the indicated primary antibodies including mouse anti sea urchin KIF5B (1:20; SUK4), mouse anti-human cytochrome *c* (clone 556432 at 1:350, BD PharMingen, San Diego, CA), goat anti-human γ-tubulin (SC-7396, Santa Cruz Biotechnology) and mouse anti-human LAMP-2 (1∶100). After washing, samples were incubated with the appropriate Alexa Fluor-488– and Alexa- Fluor-546/594-coupled secondary antibodies (Molecular Probes). Confocal images were taken using a Zeiss Axiovert 100 M Confocal Laser Scanning Microscope equipped with LSM 510 system (Carl Zeiss MicroImaging, Inc.).

### RNA extraction, cDNA synthesis and reverse transcription-PCR (RT-PCR)

The RNA was harvested from cell culture with RNeasy columns (QIAGEN) and cDNA synthesis was made with the TaqMan RT Kit (Roche) using oligo-(dT)_16_ primers. The PCR reactions were performed according to standard conditions with the following primers:

KIF1A-forw:GACACGCTGGTCTGAGATGA. KIF1A-rev:TGGCTTAGGCACTCCTCACT; KIF3A-forw:GACTATGCTGAGGCTGCAA. KIF3A-rev:TGTCTTTGGCCTTGCTTTC; KIF5A-forw:CAGCTTGACGACAAGGATGA. KIF5A-rev: GGTGTCCACTGACCTCCTGT; KIF5B-forw:GATGGATCGGAAGTGAGCAT. KIF5B-rev:ATCACGACCGTGTCTTCTCC; KIF5C-forw:GCAACTGGAACAGGAGAAGC.

KIF5C-rev:ACCTCACCCAAACACTCCAG. PBGD-forw: CATGTCTGGTAACGGCAATG; PBGD-rev:AGGGCATGTTCAAGCTCCTT. Porphobilinogen deaminase (PBGD; PubMed entry BC000520) was used as internal control together with the gene of interest. PCR products were size-separated on a 1,5%-agarose gel containing ethidium bromide, visualized under UV light, photographed using Polaroid film.

### Subcellular fractionation

For density gradient fractionation cells were pooled in ice-cold homogenization buffer (250 mM sucrose, 20 mM Hepes and 1 mM EDTA, pH 7.4) and lysed in a dounce homogenizer on ice. Homogenates were centrifuged and the supernatant spun down at 3000 g for 10 min at 4°C and the pellet was discarded. The supernatant was centrifuged at 17000 g for 20 min at 4°C. Iodixanol gradients were formed by sequential addition of 4, 10, 16 and 24% solutions in homogenization buffer at 25°C for 1 hour, resulting in the formation of a continuous gradient. The final pellet was resuspended in homogenization buffer and loaded onto a continuous 4–24% iodixanol gradient and centrifuged at 20000 *g* in a SW41Ti rotor (Beckman) for 17 h at 4°C. Gradients were separated into a total of twenty 500 μl fractions, collected from the bottom. The density of each fraction was determined by measuring OD at 244 nm. Cathepsins B/L, *N*-acetylglucosaminidase (NAG) and acidic phosphatase activities were measured for each fraction after addition of digitonin.

### Analysis of exocytosis activity upon plasma membrane wounding

Membrane wounding by electroporation was performed as described previously [21]. Briefly, cells were suspended in hanks balanced salt solution (HBSS) (Gibco, Invitrogen), subjected to electroporation at 200 V with variable levels of capacitance in a 0.2-cm electrode gene pulser cuvette (Bio-Rad), and incubated for 1 min at 37°C. Cells were then incubated with anti-LAMP-1 (sc-20011, Santa Cruz Biotechnology) antibody on ice for 30 min, washed, fixed, and stained with Alexa Fluor 488 secondary antibodies (Molecular Probes). Flow cytometry on 10000 cells per sample was performed with a FACS (Becton Dickinson) and data were analyzed by CELLQUEST software (Becton Dickinson). To measure ionomycin induced exocytosis activity cells were incubated in HBSS containing 10 μM ionomycin (Sigma). A cathepsin B specific probe, zfr-AMC (VWR International) was added to each well at a final concentration of 100 μM at time 0 and 10 min. The rate of substrate hydrolysis, as measured by the liberation of AMC (excitation wavelength, 400 nm; emission wavelength, 489 nm) at 30°C on a Spectramax Gemini fluorometer (Molecular Devices, Sunnyvale, CA, USA).

## Results

### ΔNErbB2 increases the level of KIF5B in lysosomes

**Figure 1:**
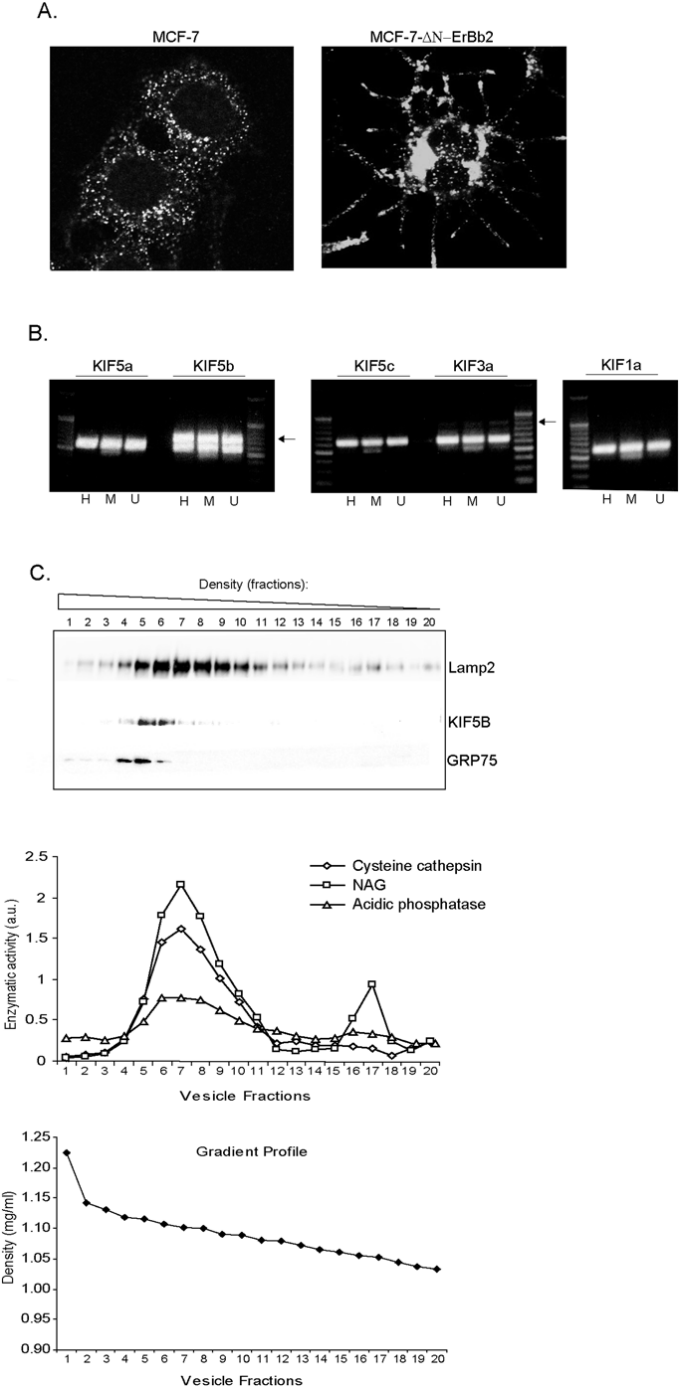
KIF5B is highly expressed in cancer cells and associates with lysosome containing fractions. (A) Immunostaining with lysosome specific LAMP-1 antibody in ΔN-ErbB2 and control cells. (B) RT-PCR analysis showing the mRNA expression levels of various N-kinesins including KIF5A (349 bp), KIF5B (337 bp), KIF5C (320 bp), KIF3A (393 bp) and KIF1A (364 bp) in H: HeLa, M: MCF-7 and U: U2OS cells. KIF amplification bands are indicated with arrows. The house-keeping gene PBDG (257 bp) was used as internal control. (C) Light membrane fractions of HeLa cells were separated by iodixanol gradient ultracentrifugation and the protein expression levels of KIF5B, LAMP-2 (lysosomes) and GRP75 (mitochondria) were visualized by immunoblotting. The enzymatic activity levels of Cathepsin B/L, acidic phosphatase and NAG was measured in all fractions and served as lysosomal markers; the linearity of the iodixanol gradient profile was determined by measuring OD at 244 nm (lower graph).

**Figure 2:**
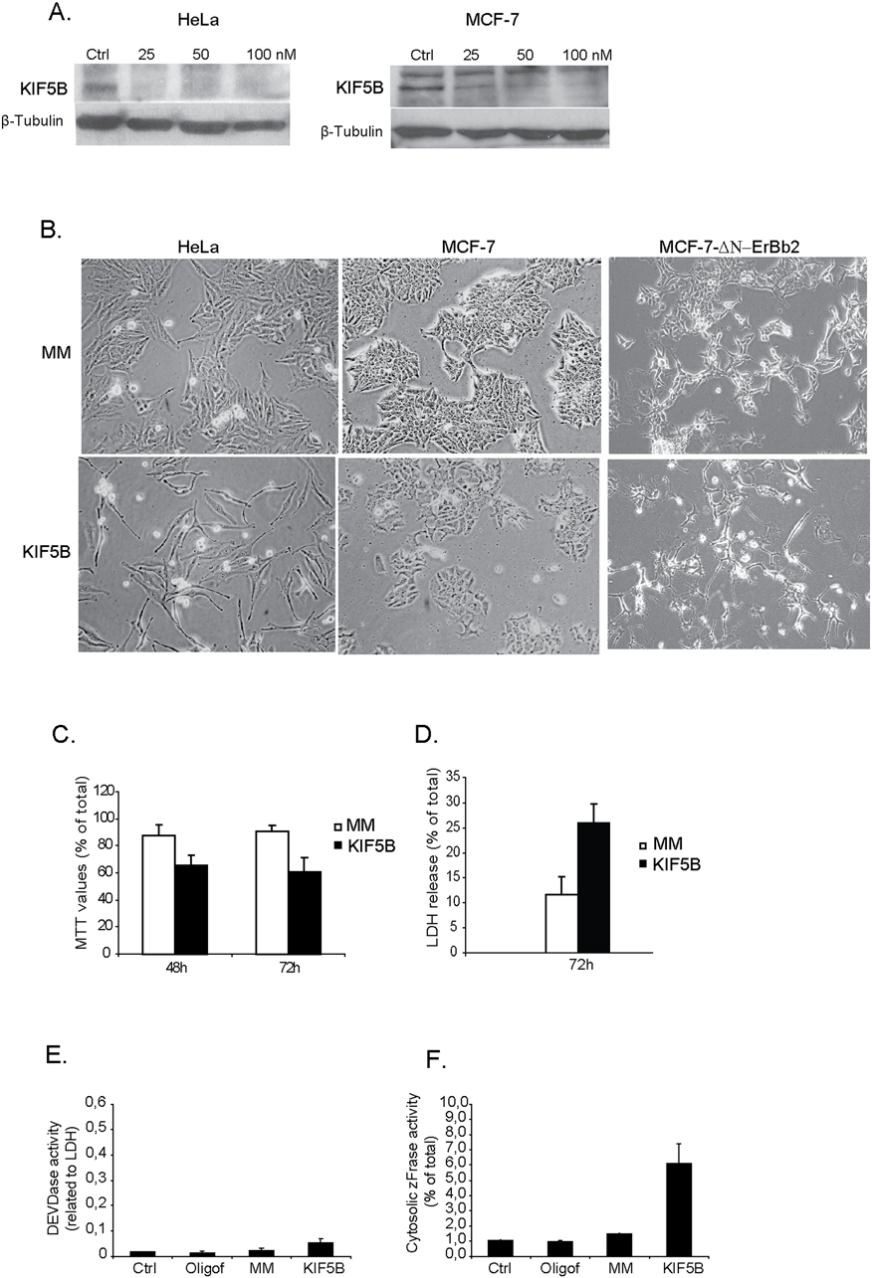
Depletion of KIF5B induces moderate cytoxicity in MCF-7 cells and massive cell death in HeLa cells. (A) Protein levels of KIF5B, 48 hours after depletion with varying siRNA concentrations (KIF5B-1 siRNA) for HeLa and MCF-7; β-tubulin served as internal control. Oligof: control cells treated with oligofectamine alone. (B) Representative phase contrast pictures of HeLa, MCF-7 and MCF-7-ΔN-ErbB2 cells, 72 h after treatment with indicated siRNAs. (C) HeLa cells were depleted for KIF5B or treated with control MM siRNA and metabolic activity determined by a MTT assay and death estimated by LDH release assay (D). MTT and LDH values is presented as percentage of untreated cells. (E) Caspase-3 like activity and (F) cytosolic cathepsin activity in HeLa cells after KIF5B depletion (72 hours) measured by DEVDase and zFRase enzyme assays respectively. Values represent means of triplicate measurements ± SD. All experiments were repeated three times with essentially the same results.

MCF-7 breast carcinoma cells expressing amino-terminally truncated constitutively active form of ErbB2 receptor tyrosine kinase (ΔNErbB2) display a highly motile phenotype characterized by extensive membrane ruffling, plasma membrane projections and scattering of the cells (Fig. 1A). Furthermore, ΔNErbB2 expression induces the localization of lysosomes to the filopodia (Fig. 1A) and a 3-4-fold up-regulation of lysosomal cysteine cathepsin activity [6] suggesting that ΔN-ErbB2 changes the lysosomal trafficking and content. In order to identify motor proteins involved in lysosomal trafficking in cancer cells, we compared the proteomes of lysosomes isolated from control MCF-7 and MCF-7-ΔNErbB2 cells by stable-isotope labeling by amino acids (Lys0/Lys8) in cell culture (SILAC) followed by mass spectrometry analysis [22]. Six myosin motors and one microtubule specific kinesin motor could be detected by this approach as lysosome-associated motor proteins (Table 1 and Dataset S1). The lysosomal association of three of the identified motor proteins (Myosin Ib, Myosin Ic and kinesin heavy chain KIF5B) was up-regulated by more than 25% upon ectopic ΔN-ErbB2 expression in MCF-7 cells. To characterize the functional significance of these three motors with regard to growth, survival and lysosomal distribution we depleted them in MCF-7 and HeLa cervix carcinoma cells by RNA interference. Only the siRNAs specific for KIF5B affected these parameters (Fig. 2 and data not shown). Thus, we chose to study the role of KIF5B on lysosomal function in more detail.

**Table 1:** Motor proteins associated with lysosomes in MCF-7-ΔN-ErbB2 compared to control MCF-7 cells as quantified by SILAC mass spectrometry.

	SWISSPROT	Ratio: ΔN-ErbB2/control
Myosin 1c	(O00159)	1,803
Myosin 1b, isoform 1	(O43795-1)	1,464
Kinesin heavy chain	(P33176)	1,372
Myosin light polypeptide 6	(P60660-2)	1,212
Myosin-9	(P35579)	1,160
Myosin-10	(P35580)	1,017
Myosin-6, isoform 2	(Q9UM54-2)	1,005

The ratio represents the average value from at least two quantified peptides per protein and indicates the relative protein abundance in ΔN-ErbB2 cells as compared to control.

### KIF5B is highly expressed in various cancer cells

First, we examined the mRNA expression levels of KIF5B in three cancer cell lines (MCF-7, HeLa and U2OS osteosarcoma) and found it to be highly expressed in all three cell lines when compared to other N-kinesins including KIF5A/KIF5C (Kinesin 1), KIF3A (Kinesin 2) and KIF1A (Kinesin 3) (Fig. 1B). We detected only low levels of KIF3A whereas both KIF5A and KIF5C mRNAs were non-detectable in all three cell lines, in agreement with earlier findings suggesting that their expression is limited to neurons [23] (Fig. 1B). In order to challenge the lysosomal localization of KIF5B detected by proteomic analysis of MCF-7 cells, we subjected the light membrane fraction of HeLa cells to a density gradient fractionation and analyzed the different fractions by immunoblotting and lysosomal enzymatic activity measurements. As shown in Figure 1C, KIF5B was exclusively present in fractions containing high levels of lysosomal LAMP-2 protein and lysosomal enzymatic activity markers (cysteine cathepsins, acidic phosphatase and ß-*N*-acetyl-glucosaminidase ). It should, however, be noted that fractions containing mitochondrial GRP75 marker protein were partly overlapping with lysosomal fractions and KIF5B.

### Depletion of KIF5B induces lysosomal leakage and cell death in HeLa cells

To elucidate the role of KIF5B in cell growth and survival, HeLa and MCF-7 cells were depleted for KIF5B by RNA interference (Fig. 2A). Interestingly, KIF5B-depleted HeLa cells acquired an elongated cellular phenotype followed by significant growth inhibition and cell death (Fig. 2B–D). KIF5B depletion induced similar but clearly weaker cytostatic/cytotoxic effects in MCF-7 cells and slightly more in MCF-7-ΔN-ErbB2 cells as analyzed by light microscopy (Fig. 2B). In spite of the induction of significant cell death, only very low caspase-3 like activity was detected in KIF5B-depleted HeLa cells (Fig. 2E). Instead, the KIF5B depleted cells displayed a significant increase in cytosolic cysteine cathepsin activity indicative of lysosomal membrane permeabilization (Fig. 2F).

### Depletion of KIF5B in HeLa cells induces peripheral aggregations of lysosomes in HeLa cells

**Figure 3:**
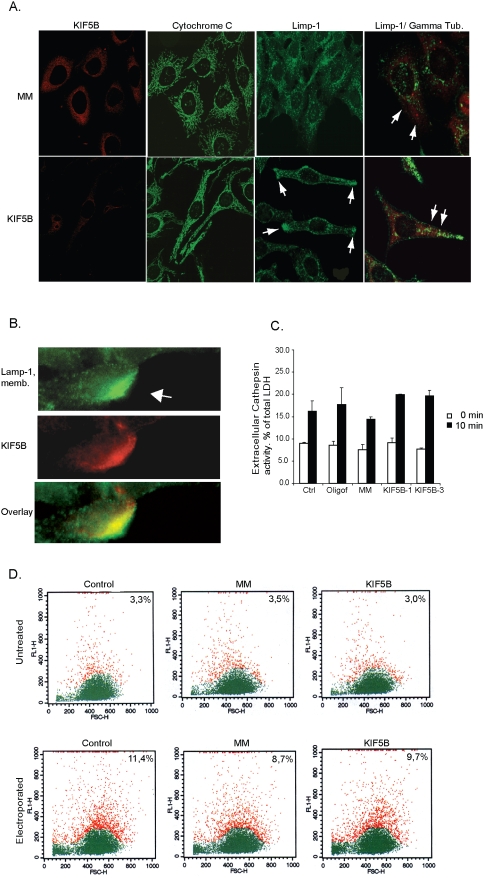
KIF5B depletion induces pericellular aggregation of lysosomes in HeLa cells but has no impact on exocytosis activity. (A) Representative confocal pictures of either HeLa cells or HeLa stably expressing LIMP-1-EGFP transfected with KIF5B or MM siRNA, and stained with indicated antibodies. (B) HeLa cells seeded on coverslips (80% confluency) were membrane wounded with a scalpel and immediately after stained for surface LAMP-1; cells were subsequently fixated and stained for KIF5B. (C) HeLa cells transfected with indicated siRNAs were (after 48 h) stimulated to exocytose with 10 μM ionomycin. Extracellular secretion of lysosomal cathepsins was measured by a zFR-AMC enzyme assay and values (means of triplicate measurements ± SD) were expressed as percent of total cellular LDH content. (D) Quantification of surface LAMP-1 in electroporated HeLa cells by flow cytometry. Red and green indicates cells in two different gates. The percentage of cells in the red gate was used to estimate the amount of surface-exposed LAMP-1 +/− electroporation. FL1-H: fluorescence intensity. FSC-H: forward side scatter.

Since KIF5B deficient mouse extraembryonic cells display perinuclear clustering of mitochondria and decreased acid-triggered trafficking of lysosomes towards the cell periphery [16], we next studied the distribution of these organelles after KIF5B depletion. In order to investigate the lysosomal distribution, we took advantage of HeLa cells expressing an eGFP-LIMP1 as a lysosomal marker [19]. In KIF5B-depleted HeLa-eGFP-LIMP1 cells the distribution of eGFP-LIMP1-postive lysosomes was dramatically altered from a diffuse perinuclear pattern to large peripheral aggregates (Fig. 3A). These lysosomes appeared in clusters and were actively transported to and from the aggregates as observed by video time-lapse microscopy (Video S1 and S2). Contrary to the lysosomes, the mitochondrial distribution was not affected by KIF5B depletion in HeLa cells (Fig. 3A).

Since KIF5B functions as a *plus*-end motor, i.e. a motor that transports cargo from the centrosome to the cell periphery [24], the accumulation of lysosomes to the cell periphery in KIF5B-depleted cells could be due to a peripheral localization of the centrosome or a failure of the lysosomes to fuse with the plasma membrane. In order to test the first possibility, we stained HeLa-eGFP-LIMP1 cells with an antibody against γ-tubulin to mark the centrosomes. However, the lysosomal clusters did not accumulate around the centrosomes (Fig. 3A). To examine if KIF5B is essential for lysosomal exocytosis, we applied three different methods (mechanical scratching, electroporation and ionomycin) to induce plasma membrane lesions that trigger Ca^2+^ influx and induction of the resealing response that involves exocytosis of lysosomes [25]. A scalpel was used to scratch on a semi-confluent layer of HeLa cells to mechanically induce plasma membrane damage, and lysosomal exocytosis was immediately assayed using an antibody detecting a luminal epitope of lysosomal LAMP-1 on the cell surface. The surface fluorescence of LAMP-1 was significantly increased at the damage site indicative of lysosomal membrane resealing, and an additional co-localization and accumulation of KIF5B at the damage site was observed suggesting that KIF5B is involved in this response (Fig. 3B). Since this method is not suitable for quantitative studies, we used electroporation to induce small hydrophilic pores in the plasma membrane to investigate if KIF5B was essential in the transport process of lysosomes to the damage sites. The method is widely used to introduce proteins and DNA into cells and depends on the cells ability to reseal their plasma membrane after electroporation [26]. HeLa cells were electroporated with increasing capacitance and immediately after they were stained for surface LAMP-1 (Fig. 3D). Quantification of LAMP-1 exposed on the plasma membrane by flow cytometry revealed a detectable level of LAMP-1 on 3,3% of untreated cells. In contrast, when cells were electroporated at 125 and 250 μF, LAMP-1 was detected on the surface in 11,4 and 21% of the cells respectively. Cells depleted for KIF5B and exposed to 125 μF did not display any significant change in surface LAMP-1 as compared to control treated cells exposed to 125 μF (Fig. 3D). Similarly, the ionomycin-induced lysosomal exocytosis of luminal proteases was unaffected by KIF5B depletion (Fig 3C). These data demonstrate that KIF5B is not crucial for the lysosomal exocytosis and plasma membrane resealing. Furthermore, the uptake of Alexa Flour 488-Dextran (10 kDa) was not affected by KIF5B depletion indicating that KIF5B is not required for fluid phase endocytosis (data not shown).

### Depletion of KIF5B induces peri-nuclear accumulation of autophagosomes

**Figure 4:**
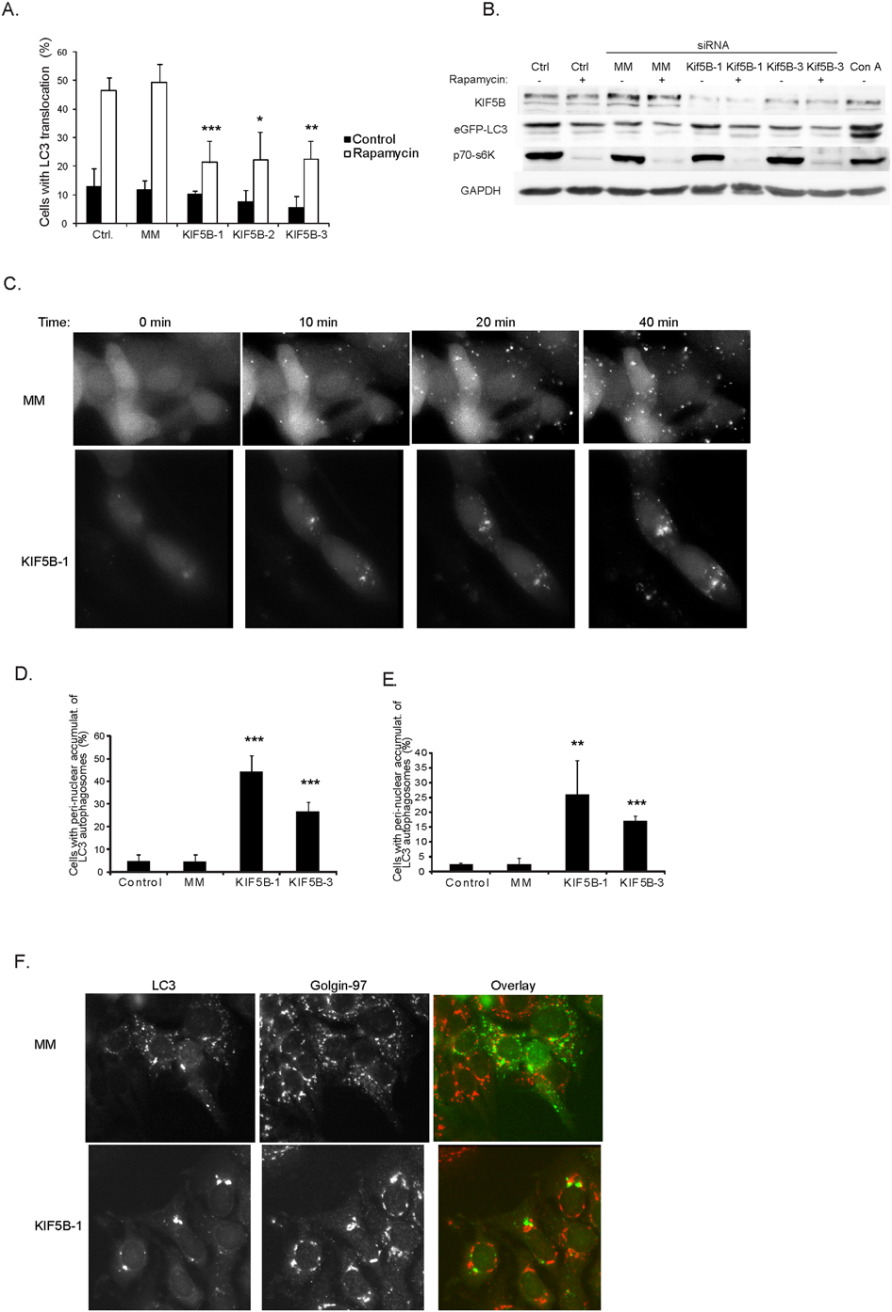
KIF5B depletion suppresses autophagy and induces nuclear accumulation of autophagosomes. (A) MCF-7-LC3-eGFP cells treated with oligofectamine or transfected with indicated siRNAs were left untreated or stimulated with rapamycin for 24 h. The percentage of cells with LC3-eGFP localized to ≥five cytosolic granular structures was estimated by counting a minimum of 100 cells/sample. Values represent means of three independent experiments ± SD. (B) Immunoblots showing the protein levels after depletion (72 h) with indicated siRNAs or treatment with 1 μM rapamycin for 12 h. p70-s6K: phosphorylated form of p70 S6 kinase. eGFP-LC3: eGFP antibody specific for eGFP (fused to LC3) (C) Representative phase contrast pictures adapted from time lapse movies of MCF-7-LC3-eGFP cells treated with Concanamycin A (6 nM) to induce autophagy and LC3-eGFP translocation to autophagosomes, 72 h after transfection with indicated siRNAs. (D and E) MCF-7-LC3-eGFP cells transfected with indicated siRNAs were (after 72 h) imaged by time lapse microscopy during treatment with either 6 nM concanamycin A (D) or 4 μM rapamycin (E). The percentage of cells displaying nuclear accumulation of LC3-eGFP autophagosomes were scored after 45 min incubation. (F) Immuno staining of *trans*-Golgi (Golgin-97 Ab) in MCF-7-LC3-eGFP cells depleted for KIF5B and incubated for 45 min with concanamycin A. Values represent means of 3–4 independent experiments. P-values: MM/KIF5B. *: p<0,05; **: p<0,01; ***: p<0,001 (student's T-test).

Next, we examined if KIF5B plays a role in autophagy, the major lysosomal degradation pathway. For this purpose, we treated MCF-7 cells stably expressing the autophagosome-associated LC3 protein fused to the enhanced green fluorescence protein (MCF-7-LC3-eGFP) with either rapamycin that induces autophagy by inactivating the mammalian target of rapamycin complex 1 (mTORC1) or concanamycin A that inhibits the vacuolar V-ATPase activity in lysosomes resulting in reduced turnover of autophagosomes as well as induction of autophagosome formation via inhibition of mTORC1 [27]. Interestingly, the depletion of KIF5B by three non-overlapping siRNAs significantly decreased the ability of rapamycin to trigger the formation of LC3-postive autophagic vesicles (Fig. 4A). This effect was not brought about by changes in the ability of rapamycin to inhibit mTORC1 since the depletion of KIF5B did not have any influence on mTORC1 activity as analyzed by the phosphorylation status of p70 S6 kinase 1 (p70^S6K1^) (Fig. 4B). To explore the phenomenon further, we followed the autophagosome formation in MCF-7-LC3-eGFP cell treated with rapamycin (not shown) or concanamycin A by time lapse video microscopy for 45 min. Surprisingly, the distribution of autophagosomes was dramatically altered by KIF5B depletion. In KIF5B depleted cells the autophagosomes appeared and accumulated mainly around the nucleus (Fig. 4C–E; Video S3) whereas in cells treated with control siRNA they were distributed diffusely throughout the cytoplasm (Fig. 4C; Video S4). The perinuclear autophagosomes in KIF5B depleted cells were located in close proximity to the golgi apparatus as visualized by staining with an antibody against a *trans*-Golgi network membrane protein Golgin-97 (Fig. 4F and Video S5). Since the distribution of Golgi was not affected by KIF5B depletion, these data suggest that KIF5B may transport a component(s) involved in the formation and/or localization of autophagosomes in the cytoplasm.

## Discussion

The data presented here suggest that KIF5B is implicated in several pathways involving lysosomes and that its highly expressed in all cancer cell lines tested, as compared to other N-Kinesins including KIF5A/KIF5C (Kinesin 1 family), KIF3A (Kinesin 2 family) and KIF1A (Kinesin 3 family). The three KIF5 subfamily members (KIF5A, KIF5B and KIF5C) display high similarity in the amino acid sequence and probably share functional redundancy and similar properties. However, at present it is unknown how the individual KIF5 members might contribute or co-operate in various transport mechanisms and why they differ in their expression patterns in neurons and non-neuronal cells. Density fractionation methodology from HeLa cells revealed, that KIF5B is mainly represented in light-membrane fractions containing lysosomes and to some extent in mitochondria.

MCF-7 and MCF-7-ΔN-ErbB2 cells depleted for KIF5B were inhibited in their growth but displayed a less pronounced death phenotype as observed in HeLa cells. Moreover, we were unable to detect any changes in lysosomal or mitochondrial distribution in MCF-7 cells, whereas HeLa cells displayed a distinct change in lysosomal distribution followed by significant death. This discrepancy between the two cell lines may be ascribed to differences in motor protein expression levels and HeLa cells are probably more dependent on KIF5B for plus-end directed motor protein activity. The aggregation of lysosomes in the pericellular area preceding death in HeLa cells suggested that KIF5B might play a role in trafficking lysosomes proximal to the plasma membrane. However, we were unable to detect any reduction in exocytosis activity triggered either by ionomycin, or electroporation induced membrane damage, following KIF5B depletion. Nevertheless, a significant KIF5B translocation was observed, when HeLa cells were exposed to mechanical induced plasma membrane lesions to induce the resealing response and facilitate lysosomal exocytosis. Here, we observed a considerable recruitment of LAMP-1 positive lysosomes to reseal the damage site and significant colocalization with KIF5B. These data signify that KIF5B could play a role in transporting lysosomes to the plasma membrane destined for exocytosis, but functional redundancy probably exists that implicates other motor proteins as well. The aggregation of lysosomes following KIF5B depletion can also be explained by an alternative possibility: that KIF5B besides its role as a transporter plays a role in positioning lysosomes at distinct sites in the cytoplasm (predominantly perinuclear). Accordingly, depletion of KIF5B would liberate lysosomes allowing their transport by other motor proteins including N-kinesins towards the cortical areas of the cell resulting in lysosomal aggregations. This implies that recruitment of lysosomes by KIF5B to microtubules may localize or queue them and not necessarily facilitate long distance travel.

The death pathway induced after KIF5B depletion in HeLa cells triggered the aggregation of lysosomes followed by lysosomal destabilization and subsequent release of lysosomal cathepsins to the cytosol. We observed only limited caspase-3 like activation suggesting that the classical caspase mediated death pathway plays a minor role in the death mode observed. Lysosomal cathepsins function as effective mediators of programmed cell death but the pathways leading to LMP are, however, poorly understood. We have recently shown that vincristine, a compound that destabilizes microtubules and is frequently used in cancer therapy, induce dramatic aggregations of lysosomes and induces LMP and death in HeLa cells [10]. The two treatments might have similar consequences for lysosomal distribution resulting in lysosomes that are brought together in an uncontrolled manner inducing aggregations and subsequent destabilization of lysosomes resulting in death.

The more pronounced death phenotype observed in HeLa cells when compared to MCF-7/MCF-7-ΔN-ErbB2 cells could be explained by a higher dependency on proper KIF5B motor protein activity basically to deal with a high metabolic activity and growth rate. Alternatively, MCF-7 cells might encompass more functional redundancy through expression of various motor proteins than in HeLa cells. Most of the knowledge about KIF5 mediated transport is based on studies in neurons where KIF5 family members can transport various cargoes and its activity seems to be dominant over other motor activities [12], however the data in non-neuronal cells are still limited.

Depletion of KIF5B by RNA interference in MCF-7 cells had a surprising impact on autophagosome formation/localization as determined by the translocation of LC3-eGFP to autophagosomes. We estimated autophagic activity by scoring LC3-eGFP positive autophagosomes after stimulation with rapamycin and observed a significant reduction upon KIF5B depletion. This finding prompted us to follow autophagosome formation in real time by time lapse microscopy using higher concentrations of either rapamycin or concanamycin A enabling us to follow the process in a shorter time frame. The remarkable accumulations of autophagosomes around the nucleus in cells depleted for KIF5B suggested that KIF5B could be involved directly in the transport of autophagosomes along microtubule tracks to the cytoplasm. However, our recent quantitative mass-spectometry analysis on membrane associated proteins purified from autophagosomes (from MCF-7 cells treated with rapamycin or concanamycin A) revealed that KIF5B is not directly associated with autophagosomes (MHH, JA, JO, unpublished data). Alternatively, KIF5B might be involved in the transport of critical factor(s) important for initiation of the autophagic process and proper distribution of autophagosomes in the cytoplasm. Accordingly, removal of KIF5B might prevent the transport of initiation factor(s) and result in formation of autophagosomes accumulating close to the microtubule organizing center and nucleus. Since the autophagosomes appeared mainly at one site of the nucleus (Fig. 4C and Video S3) and close to the *trans*-golgi network (Fig. 4F and Video S5) suggests, that the putative initiation factor(s) transported by KIF5B could be golgi derived and affect the distribution of autophagosomes after KIF5B depletion. In addition, KIF5B depletion did not have any influence on the activity of mTOR (as determined by the phosphorylation status of p70 S6 kinase) indicating that the motor protein is acting down-stream of mTOR.

Our data demonstrate that KIF5B is highly expressed in cancer cells and plays a significant role in growth and survival of HeLa cells. Additionally, we show that KIF5B is very abundant at the site of plasma membrane damage co localizing with lysosomes destined for exocytosis although it's not essential suggesting that functional redundancy probable exist. Moreover, we provide data indicating that KIF5B is involved in the initial formation/localization of autophagosomes and might transport component(s) important for the autophagic process.

## Supporting Information

Dataset S1 Motor proteins associated with lysosomes in MCF-7-deltaN-ErbB2 compared to control MCF-7 cells as quantified by SILAC mass spectrometry.
(1.32 MB XLS)

Video S1 Control of HeLa-LIMP1-eGFP cells
(1.58 MB MOV)

Video S2 HeLa-LIMP1-eGFP cells depleted for KIF5B (after 72h) and imaged by time-lapse microscopy.
(8.62 MB MOV)

Video S3 MCF-7-LC3-eGFP cells depleted for KIF5B were stimulated with 6 nM Concanamycin A and followed by time-lapse microscopy.
(8.90 MB MOV)

Video S4 MCF-7-LC3-eGFP cells treated with control siRNA (MM, 72h) were stimulated with 6 nM Concanamycin A and followed by time-lapse microscopy.
(9.23 MB MOV)

Video S5 MCF-7-LC3-eGFP cells co-expressing dsRED-Golgi (trans Golgi stack) and depleted for KIF5B were stimulated with 6 nM Concanamycin A and imaged by time-lapse microscopy.
(8.48 MB MOV)
